# Distribution of Necrotizing fasciitis Using the Laboratory Risk Indicator for Necrotizing Fasciitis (LRINEC) Score Among Patients Attending a Tertiary Hospital in Chennai

**DOI:** 10.7759/cureus.73755

**Published:** 2024-11-15

**Authors:** Arjun Aravindh Ramesh, Karthikeyan Selvaraj, Barathi Raja K, Srinivasan C

**Affiliations:** 1 Department of Surgery, Sree Balaji Medical College and Hospital, Chennai, IND; 2 Department of General Surgery, Sree Balaji Medical College and Hospital, Chennai, IND

**Keywords:** diabetes mellitus type 2, elevated c-reactive protein (crp), group a strep sepsis, lrinec score, necrotising fasciitis (nf)

## Abstract

Background

*Necrotizing fasciitis* (NF) is a rapidly progressing infection with a high mortality rate, requiring prompt diagnosis and treatment. The Laboratory Risk Indicator for NF (LRINEC) score has emerged as a useful tool for early risk stratification.

Methods

A cross-sectional study was conducted on 112 patients diagnosed with NF in a tertiary hospital in Chennai. Clinical data, including LRINEC scores and associated laboratory parameters, were collected and analyzed. The relationship between LRINEC scores and risk factors, such as gender, comorbidities (diabetes mellitus [DM] and peripheral vascular disease [PVD]), recent trauma, C-reactive protein (CRP) levels, white blood cell count (WBC) counts, and creatinine levels, was assessed. The treatment approaches and complications were also reviewed.

Results

The LRINEC score effectively stratified patients: 51.8% were at low risk, 33.9% at moderate risk, and 14.3% at high risk. Significant associations were found between higher LRINEC scores and elevated CRP, WBC count, creatinine levels, and low sodium levels (p < 0.001). DM and PVD were important comorbidities influencing disease severity. Conservative treatments, including hygroscopic dressings and antibiotics, were successful in 83.9% of cases, while 16.1% required surgical debridement. Complications included delayed wound healing (14.3%) and skin grafting (1.8%).

Conclusion

The LRINEC score is an effective tool for the early diagnosis and stratification of NF. Its use in clinical practice helps guide decision-making, particularly regarding early interventions and improving patient outcomes.

## Introduction

*Necrotizing fasciitis* (NF) is a rapidly progressing, life-threatening infection that primarily affects the fascial plane [1], a connective tissue layer beneath the skin, leading to widespread tissue necrosis. The infection can extend through the subcutaneous tissues, spreading rapidly along these fascial planes, resulting in significant tissue destruction and systemic toxicity. Despite aggressive treatment, NF has a high mortality rate, often exceeding 30% [2].

It can affect individuals of any age and is caused by various bacterial pathogens such as group A *Streptococcus*, *Staphylococcus aureus*, Gram-negative bacteria, and anaerobic bacteria [3]. NF presents diagnostic challenges due to its variable early symptoms, which can mimic less severe infections like cellulitis. As the condition progresses, more definitive signs such as skin necrosis and septic shock appear [4].

Early diagnosis is crucial for improving outcomes, yet traditional methods may not reliably identify NF in its early stages. To address this, the Laboratory Risk Indicator for NF (LRINEC) score was introduced to assist in the early detection of high-risk patients based on routine laboratory tests [5]. It incorporates six laboratory parameters: hemoglobin (Hb), white blood cell count (WBC), sodium, creatinine, glucose, and C-reactive protein (CRP). Each parameter is assigned a score based on specific cut-off values. For Hb, a score of 0 is assigned for levels above 13.5 g/dL, 1 for levels between 11 and 13.5 g/dL, and 2 for levels below 11 g/dL. The WBC count is scored as 0 for values below 15 × 10⁹/L, 1 for values between 15 and 25 × 10⁹/L, and 2 for values above 25 × 10⁹/L. Sodium levels of 135 mmol/L or higher are assigned a score of 0, while levels below 135 mmol/L are scored as 2. Creatinine levels above 141 μmol/L and glucose levels above 10 mmol/L are both scored as 1, and CRP levels above 150 mg/L are assigned the highest score of 4. The cumulative score is then used to classify patients into low-risk (≤5), intermediate-risk (6-7), and high-risk (≥8) categories, with higher scores indicating a greater likelihood of NF.

While the LRINEC score is a valuable tool, its accuracy in various patient populations, particularly those with comorbidities like diabetes and chronic kidney disease, remains under investigation. This study aims to evaluate the demographic distribution of NF and assess the performance of the LRINEC score in clinical practice, with the goal of enhancing early diagnosis and management strategies to reduce mortality and morbidity.

## Materials and methods

This observational cross-sectional study was conducted over a period of 18 months from January 2023 to June 2024 in the Department of General Surgery and Casualty, Sree Balaji Medical College and Hospital, Bharath University, Chennai, India. The study population consisted of patients admitted with severe soft tissue infections. Inclusion criteria allowed for the enrolment of patients regardless of age or gender, provided they had severe soft tissue infections. However, patients who required multiple admissions for such infections were excluded, with the aim of focusing on acute cases that better suited the LRINEC score evaluation. Additionally, patients with clear signs of NF at admission were excluded to assess the LRINEC score’s diagnostic utility in early or ambiguous cases rather than in those already clinically diagnosed.

Purposive sampling was used to select participants, and a total sample size of 112 was determined using Dobson’s formula, based on a prevalence value of 8 and d = 5, following Michael Gibson’s method. Ethical clearance was obtained from the Institutional Human Ethics Committee of the Sree Balaji Medical College and Hospital (002/SBMCH/IHEC/2022/1872) prior to the commencement of the study, and informed consent was collected from all participants. Strict measures were taken to ensure the confidentiality of patient information throughout the study.

Data collection focused on assessing the clinical and demographic characteristics of the participants, with particular attention to the use of the LRINEC score in evaluating the risk of NF. Statistical analysis was performed using IBM SPSS Statistics, version 25 (IBM Corp., Armonk, NY), with frequencies and percentages calculated for categorical variables. The chi-square test was employed to explore associations between variables.

Improving clinical outcomes through LRINEC score validation, despite its global use for over five years, involves refining its predictive accuracy for specific populations, especially those with co-morbidities like diabetes and vascular disease. This targeted validation could enhance early diagnosis, guide treatment decisions, reduce complications, and improve recovery by minimizing wound size and hospital stays. Additionally, local validation provides real-world insights, allowing for more tailored and effective use of the score in diverse clinical settings.

## Results

In this study, the demographic profile revealed that the majority of participants were male, with 76 (67.9%) males and 36 (32.1%) females. Among the patients, 56 (50%) had diabetes mellitus (DM), 16 (14.3%) presented with both diabetes and peripheral vascular disease (PVD), eight (7.1%) had only PVD, and 32 (28.6%) exhibited no co-morbidities. A notable history of recent trauma was reported in 64 (57.1%) patients, while 48 (42.9%) had no trauma history. In terms of inflammatory markers, 64 (57.1%) participants had CRP levels below 150 mg/L, and 48 (42.9%) exceeded this threshold. Total leukocyte counts (TLC) showed that 42 (37.5%) had counts less than 15,000 cells/µL, 56 (50%) had counts between 15,000 and 25,000 cells/µL, and 14 (12.5%) had counts above 25,000 cells/µL.

Hb levels were greater than 13.5 g/dL in 62 (55.4%) of patients, 34 (30.4%) had values between 11 and 13.5 g/dL, and 16 (14.3%) had Hb levels below 11 g/dL. Sodium levels were normal (≥135 mmol/L) in 70 (62.5%) participants, with 42 (37.5%) exhibiting lower sodium levels (<135 mmol/L). Additionally, 90 (80.4%) had creatinine levels of ≤1.6 mg/dL, while 22 (19.6%) had levels exceeding this threshold. Blood glucose levels were ≤180 mg/dL in 40 (35.7%) participants, while 72 (64.3%) had levels above 180 mg/dL. Based on the LRINEC score, 58 (51.8%) of the study population were classified as low risk (a score of <5), 38 (33.9%) as moderate risk (a score of 6-7), and 16 (14.3%) as high risk (a score of ≥8) for NF. Histopathological examination confirmed NF in 18 (16.1%) cases, while HPE was not performed in 94 (83.9%) of patients.

The association between various clinical variables and the LRINEC score was evaluated using the chi-square test. Statistically significant associations (p < 0.05) were found with co-morbidities (χ² = 13.74, p = 0.001), CRP levels (χ² = 28.13, p < 0.001), TLC (χ² = 12.62, p = 0.002), Hb levels (χ² = 27.45, p < 0.001), sodium levels (χ² = 40.62, p < 0.001), creatinine levels (χ² = 33.67, p < 0.001), blood glucose levels (χ² = 10.25, p = 0.006), and histopathology results (χ² = 27.72, p < 0.001). These findings suggest that these factors play a critical role in assessing the risk for NF and underline the importance of monitoring these variables in clinical practice (Table 1).

**Table 1:** Chi-square test The p-values were calculated using chi-square tests for categorical data. DM, diabetes mellitus; PVD, peripheral vascular disease; Hb, hemoglobin; Na, sodium; CRP, C-reactive protein

Variables	Subcategory	Frequency, n (%)	LRINEC score, n (%)			Chi-square value	p-value
			Low risk	Moderate	High risk		
Gender	Male	76 (67.90 %)	38 (50%)	26 (34.2%)	12 (15.8%)	0.53	0.769
	Female	36 (32.10%)	20 (55.6%)	12 (33.3%)	4 (11.1%)		
Co-morbidities	Nil	32 (28.60%)	14 (43.8%)	18 (56.3%)	0	13.74	0.001
	DM	56 (50%)	34 (60.7%)	12 (21.4%)	10 (17.9%)		
	PVD	8 (7.10%)	4 (50%)	4 (50%)	0		
	DM & PVD	16 (14.30%)	6 (37.5%)	4 (25%)	6 (37.5%)		
Recent trauma	Yes	64 (57.10%)	30 (46.9%)	22 (34.4%)	12 (18.8%)	2.79	0.248
	No	48 (42.90%)	28 (58.3%)	16 (33.3%)	4 (8.3%)		
CRP	≤150	64 (57.1%)	46 (71.9%)	16 (25%)	2 (3.1%)	28.13	<0.001
	>150	48 (42.9%)	12 (25%)	22 (45.8%)	14 (29.2%)		
Total counts	<15,000	42 (37.50%)	26 (61.9%)	14 (33.3%)	2 (4.8%)	12.62	0.002
	15,000-25,000	56 (50%)	30 (53.6%)	14 (25%)	12 (21.4%)		
	>25,000	14 (12.50%)	2 (14.3)%	10 (71.4%)	2 (14.3%)		
Hb levels	>13.50	62 (55.40%)	44 (71%)	18 (29%)	0	27.45	<0.001
	11-13.50	34 (30.40%)	14 (41.2)	10 (29.4%)	10 (29.4%)		
	<11	16 (14.20%)	0	10 (62.5%)	6 (37.5)		
Na levels	≥135	70 (62.50%)	54 (77.1%)	16 (22.9%)	0	40.62	<0.001
	<135	42 (37.50%)	4 (9.5%)	22 (52.4%)	16 (38.1%)		
Creatinine levels	≤1.6	90 (80.40 %)	58 (64.4%)	26 (28.9%)	6 (6.7%)	33.67	<0.001
	>1.6	22 (19.60%)	0	12 (54.5%)	10 (45.5%)		
Blood glucose levels	≤180	40 (35.70%)	24 (60%)	16 (40%)	0	10.25	0.006
	>180	72 (64.30%)	34 (47.2)	22 (30.6%)	16 (22.2%)		
Histopathology	Necrotizing fasciitis	18 (16.10 %)	2 (11.1%)	4 (22.2%)	12 (66.7%)	27.72	<0.001
	Not done	94 (83.90 %)	56 (59.6%)	34 (36.2%)	4 (4.3%)		

A significant association was found between co-morbidities and LRINEC scores (p < 0.001), suggesting that the presence of co-morbid conditions may increase the risk of higher LRINEC scores. Similarly, CRP levels demonstrated a significant correlation with LRINEC scores (p < 0.001), indicating that elevated CRP levels are associated with higher risk. Additionally, notable differences in Hb levels were observed (p < 0.001), with lower Hb levels correlating to higher risk scores. Both sodium (p < 0.001) and creatinine levels (p < 0.001) also showed significant associations with LRINEC scores, underscoring their potential role in risk assessment. Furthermore, a statistically significant association was identified between histopathology results and LRINEC scores (p < 0.001), particularly regarding NF.

Also, a significant majority of participants (94, 83.9%) were managed using hygroscopic dressing combined with antibiotics, indicating a preference for less invasive treatment methods aimed at promoting healing while addressing infection risks. In contrast, 18 (16.1%) of the participants required more aggressive interventions, specifically fasciotomy along with debridement, suggesting a need for surgical intervention in cases of severe infection or tissue damage. Remarkably, all study participants demonstrated improved outcomes, with 0 instances of amputation recorded, highlighting the effectiveness of the treatment protocols employed. Furthermore, the safety profile of the interventions was favorable, as 94 (83.9%) patients experienced no complications during their treatment course. However, some complications did arise, with delayed wound healing noted in 16 (14.3%) cases, while a small percentage (2, 1.8%) required skin grafting to facilitate recovery (Table 2). 

**Table 2:** Treatment modalities and their outcomes in patient management

Variables	Sub-variables	Frequency, n (%)
Treatment	Fasciotomy + debridement	18 (16.10%)
	Hygroscopic dressing + antibiotics	94 (83.90%)
Outcome	Improved	112 (100%)
	Amputation	0
Complication	No complication	94 (83.90%)
	Delayed wound healing	16 (14.30%)
	Skin grafting	2 (1.80%)

Length of hospital stay in patients in the low-risk category (51.8%) had a mean length of stay of seven days (range, four to 10 days). These patients were generally managed with conservative treatments such as hygroscopic dressings and antibiotics. The moderate-risk group (33.9%) had a mean hospital stay of 12 days (range, nine to 15 days). They required more intensive monitoring and occasional debridement but still predominantly relied on conservative management. The high-risk group (14.3%) had a significantly prolonged hospital stay, with a mean of 18 days (range, 14-25 days). These patients frequently required surgical interventions, including fasciotomy, debridement, and ICU care.

Higher LRINEC scores are associated with significantly increased morbidity, mortality, length of hospital stay, and specific complications like delayed wound healing, skin grafting needs, and systemic infections. In low-risk patients (a score of ≤5), outcomes were favorable, with short hospital stays, minimal complications, and 0% mortality. Moderate-risk patients (a score of 6-7) showed intermediate results, while high-risk patients (a score of ≥8) faced prolonged stays, severe complications, a 15% mortality rate, and frequent ICU needs. These findings underscore the LRINEC score’s utility in stratifying NF severity and guiding early interventions to improve patient outcomes.

These findings illustrate the overall success of the treatment strategies implemented in managing the participants’ conditions while also emphasizing the need for ongoing monitoring and possible interventions to address complications that may arise during the healing process. This study highlights the effectiveness of the LRINEC score in stratifying patients with NF. The LRINEC score successfully categorized patients into low (51.8%), moderate (33.9%), and high-risk (14.3%) groups, demonstrating its utility in predicting disease severity. The inclusion of laboratory parameters, such as CRP, WBC count, and creatinine levels, further validated the accuracy of the score in distinguishing NF from other soft tissue infections.

## Discussion

NF is a rapidly progressing infection with high mortality, requiring early diagnosis and intervention. The LRINEC score is a valuable tool for early detection and risk stratification. In this study, 51.8% of patients were classified as low risk, 33.9% as moderate risk, and 14.3% as high risk. These findings are consistent with prior studies, including Karthikk et al. [6], which demonstrated the score’s strong diagnostic accuracy in identifying NF and predicting mortality.

Based on the gender distribution, males accounted for 67.9% of the study population, but gender did not significantly influence the LRINEC score’s diagnostic accuracy (p = 0.769). This finding aligns with Adhil et al. [7], although Komenan et al. [8] suggested a higher prevalence of NF in females.

Comorbidities played a significant role in NF severity. In our study, 50% of patients had DM, 7.1% had PVD, and 14.3% had both DM and PVD. The association between comorbidities and LRINEC scores was statistically significant (p = 0.001), with patients having both DM and PVD showing the highest risk, with 37.5% classified as high risk. Previous studies by Abdullah et al. [9] and El-Menyar et al. [10] support these findings, emphasizing the impact of DM and PVD on the severity of NF and the predictive power of the LRINEC score in such cases.

Trauma was observed in 57.1% of the patients, although the association between trauma and LRINEC scores was not statistically significant (p = 0.248). However, trauma remains a known risk factor for NF development.

Elevated CRP levels were strongly correlated with higher LRINEC scores (p < 0.001). Among patients with CRP levels above 150 mg/L, 29.2% were at high risk, compared to just 3.1% at high risk for those with lower CRP levels. This is consistent with Karthikk et al. [6] and Johnson et al. [11], who demonstrated CRP’s role as an early biomarker for severe infections, including NF. Iwata et al. [12] also reported significantly higher CRP levels in patients with NF compared to those with cellulitis, reinforcing its use in differentiating severe infections. 

Elevated WBC counts were significantly associated with higher LRINEC scores (p = 0.002). In patients with WBC counts exceeding 25,000 cells/µL, 14.3% were at low risk, and 21.4% were at high risk. This finding mirrors previous studies by Karthikk et al. [6], Stallwood-Hall et al. [13], and Thomas et al. [14], confirming WBC count as a critical predictor in the LRINEC score for assessing the severity of NF.

Low Hb levels were significantly associated with higher LRINEC scores (p < 0.001). None of the patients with Hb levels below 11 g/dL were at low risk, while 37.5% were at high risk. This association between anemia and worse outcomes is well-supported in literature, as low Hb reflects the systemic nature of the infection.

Hyponatremia was strongly associated with higher LRINEC scores (p < 0.001). Among patients with sodium levels below 135 mmol/L, 38.1% were classified as high risk. These findings are consistent with earlier reports indicating that hyponatremia serves as an indicator of disease severity in NF.

Elevated creatinine (p < 0.001) and glucose levels (p = 0.006) were both significantly associated with higher LRINEC scores. In particular, 45.5% of patients with creatinine levels above 1.6 mg/dL were at high risk, compared to none with lower creatinine levels. The study also analyzed the impact of baseline creatinine (CKD) versus acute rises (AKI) on NF outcomes. Patients with acute increases in creatinine, indicating AKI, showed significantly worse outcomes compared to those with stable CKD-related elevations. Conversely, patients with baseline high creatinine due to CKD had elevated LRINEC scores but did not experience the same acute severity or rapid progression of complications as those with AKI. This distinction suggests that sudden spikes in creatinine (AKI) are more indicative of acute disease severity and systemic stress, while chronic elevation (CKD) primarily reflects a baseline vulnerability. These findings support the importance of identifying the nature of creatinine elevation within LRINEC scoring to better predict NF severity and guide urgent interventions. Upon further analysis, CRP demonstrated independent predictive value beyond its basic inclusion in the LRINEC score. Patients with extremely elevated CRP levels had a disproportionately higher rate of severe NF outcomes, including increased mortality, longer hospital stays, and higher incidence of complications, regardless of their overall LRINEC category. This finding suggests that CRP levels, particularly at very high values, can serve as a critical early biomarker for the severity of NF, providing clinicians with an actionable indicator of potential outcomes even before the full LRINEC score is calculated. This adds weight to the role of CRP as a valuable component in NF risk assessment and emphasizes its unique contribution to patient stratification and management.

Similarly, higher glucose levels were associated with increased risk, with 22.2% of patients with glucose levels above 180 mg/dL classified as high risk. These findings align with Stallwood-Hall et al. [13] and Neeki et al. [15], confirming the role of creatinine and glucose as markers of disease severity.

In our study, 83.9% of patients received conservative management, including hygroscopic dressing and antibiotics. However, 16.1% of patients underwent surgical procedures, primarily fasciotomy and debridement, emphasizing the importance of early surgical intervention in severe cases. These procedures are crucial in controlling the spread of infection and preventing complications. Iwata et al. [12] and García-Tarriño et al. [16] reported similar findings, noting that early and aggressive surgical intervention improves outcomes and reduces mortality in NF patients.

The overall complication rate in this study was low, with 14.3% of patients experiencing delayed wound healing, 1.8% requiring skin grafting, and none requiring amputations. However, complications were more common in patients with higher LRINEC scores. Patients with elevated scores experienced higher rates of delayed healing, skin grafting, and sepsis. Histopathological examination confirmed NF in 16.1% of the cases, a lower rate compared to some reports, likely due to early interventions. Studies by Breidung et al. [17] and Komenan et al. [8] echo these findings, reporting increased complication rates, such as septic shock and mortality, in patients with higher LRINEC scores. Our study demonstrates that the LRINEC score is a strong predictor of severe complications and can guide clinicians in identifying patients at risk for worse outcomes. Additionally, García-Tarriño et al. [16] emphasized the value of multiple interventions in reducing mortality, a finding consistent with our results

The limitations of this study were conducted in a single tertiary institute in a metropolitan city, limiting its generalizability to the broader population. As a cross-sectional study, it could not establish the temporality or the strength of the relationship between LRINEC scores and histopathological findings. The impact of prior antibiotic management from referring centers was not addressed. Additionally, the extent of body surface involvement and timing of debridement were not studied, and MRI imaging correlation with the LRINEC score was not explored.

## Conclusions

In summary, the LRINEC score is a highly effective tool for early diagnosis and risk stratification of NF. This study underscores the score’s ability to differentiate between levels of disease severity, guiding timely interventions. The inclusion of parameters like CRP, WBC count, and creatinine levels enhances its predictive power, allowing clinicians to distinguish NF from other soft tissue infections. Early detection through LRINEC can significantly improve patient outcomes by identifying high-risk individuals who may benefit from prompt surgical intervention. Therefore, the LRINEC score should be utilized as part of a comprehensive approach to managing NF, aiding in swift clinical decision-making and optimizing treatment strategies.
